# (μ-*trans*-1,2-Di-4-pyridylethyl­ene-κ^2^ *N*:*N*′)bis­[bis­(*N*,*N*-diisopropyl­dithio­carbamato-κ^2^ *S*,*S*′)zinc(II)]

**DOI:** 10.1107/S1600536809044250

**Published:** 2009-10-31

**Authors:** Hadi D. Arman, Pavel Poplaukhin, Edward R. T. Tiekink

**Affiliations:** a Department of Chemistry, The University of Texas at San Antonio, One UTSA Circle, San Antonio, Texas 78249-0698, USA; b Chemical Abstracts Service, 2540 Olentangy River Rd, Columbus, Ohio 43202, USA; c Department of Chemistry, University of Malaya, 50603 Kuala Lumpur, Malaysia

## Abstract

The dinuclear title compound, [Zn_2_(C_7_H_14_NS_2_)_4_(C_12_H_10_N_2_)], is centrosymmetric about the central C=C bond. The five-coordinate Zn atom is bonded to two asymmetrically chelating dithio­carbamate ligands and a pyridine N atom to define an NS_4_ coordination geometry tending towards a square pyramid, with the N atom in the apical site. In the crystal structure, C—H⋯S contacts lead to supra­molecular chains.

## Related literature

For background to supra­molecular polymers of zinc 1,1-dithiol­ates, see: Lai *et al.* (2002 ▶); Chen *et al.* (2006 ▶); Benson *et al.* (2007 ▶). For a related structure and the synthesis, see: Lai & Tiekink (2003 ▶). For additional geometrical analysis, see: Addison *et al.* (1984 ▶).
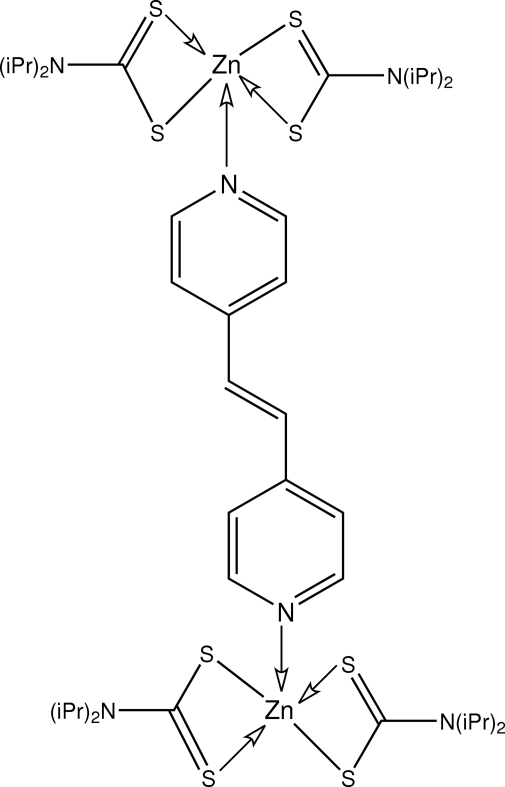


## Experimental

### 

#### Crystal data

[Zn_2_(C_7_H_14_NS_2_)_4_(C_12_H_10_N_2_)]
*M* *_r_* = 1018.21
Triclinic, 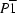

*a* = 8.2690 (14) Å
*b* = 11.1640 (18) Å
*c* = 14.156 (2) Å
α = 80.806 (10)°
β = 84.878 (9)°
γ = 72.566 (5)°
*V* = 1229.6 (3) Å^3^
*Z* = 1
Mo *K*α radiation
μ = 1.35 mm^−1^
*T* = 98 K
0.43 × 0.35 × 0.22 mm

#### Data collection

Rigaku AFC12K/SATURN724 diffractometer
Absorption correction: multi-scan (*ABSCOR*; Higashi, 1995 ▶) *T*_min_ = 0.810, *T*_max_ = 1
9453 measured reflections
5613 independent reflections
5323 reflections with *I* > 2σ(*I*)
*R*_int_ = 0.027

#### Refinement

*R*[*F* ^2^ > 2σ(*F* ^2^)] = 0.038
*wR*(*F* ^2^) = 0.103
*S* = 1.08
5613 reflections
261 parameters
H-atom parameters constrained
Δρ_max_ = 0.73 e Å^−3^
Δρ_min_ = −0.93 e Å^−3^

### 

Data collection: *CrystalClear* (Rigaku/MSC, 2005 ▶); cell refinement: *CrystalClear*; data reduction: *CrystalClear*; program(s) used to solve structure: *SHELXS97* (Sheldrick, 2008 ▶); program(s) used to refine structure: *SHELXL97* (Sheldrick, 2008 ▶); molecular graphics: *DIAMOND* (Brandenburg, 2006 ▶); software used to prepare material for publication: *SHELXL97*.

## Supplementary Material

Crystal structure: contains datablocks global, I. DOI: 10.1107/S1600536809044250/hb5178sup1.cif

Structure factors: contains datablocks I. DOI: 10.1107/S1600536809044250/hb5178Isup2.hkl

Additional supplementary materials:  crystallographic information; 3D view; checkCIF report

## Figures and Tables

**Table 1:** Selected bond lengths (Å)

Zn—N3	2.0621 (18)
Zn—S1	2.3655 (7)
Zn—S3	2.3662 (7)
Zn—S2	2.5320 (7)
Zn—S4	2.5720 (7)

**Table 2:** Hydrogen-bond geometry (Å, °)

*D*—H⋯*A*	*D*—H	H⋯*A*	*D*⋯*A*	*D*—H⋯*A*
C20—H20⋯S4^i^	0.95	2.77	3.545 (2)	139

Symmetry code: (i) 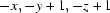.

**Fig. 1.**
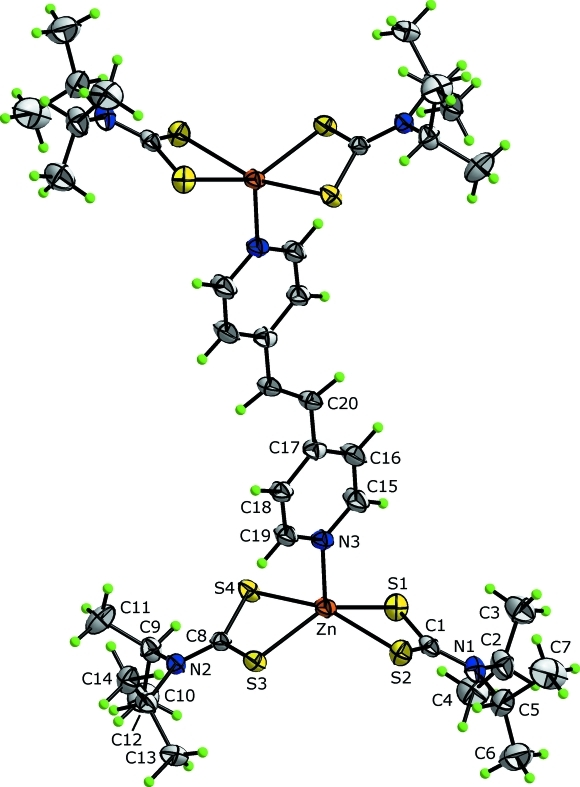
The molecular structure of (I) with the asymmetric unit labelled; unlabelled atoms are related by the symmetry operation -1 - x, 1 - y, 1 - z. Displacement ellipsoids are shown at the 70% probability level.

**Fig. 2.**
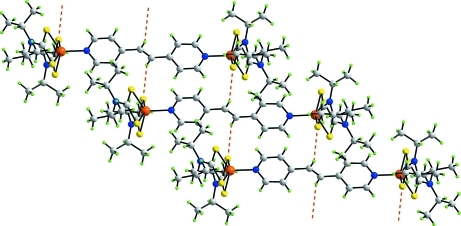
Supramolecular chain formation in (I) mediated by C—H···S contacts (orange dashed lines).

## supplementary crystallographic information

### Comment

Crystal engineering studies of zinc(II) 1,1-dithiolates (Lai *et al.*, 2002; Chen *et al.*, 2006; Benson *et al.* 2007) motivated the synthesis of the title compound (I). The dinuclear compound is centrosymmetric and features a five coordinate Zn atom. Two asymmetrically chelating dithiocarbamate ligands (range of Zn–S = 2.3655 (7) to 2.5720 (7) Å) and a pyridine-N atom (Zn–N 2.0621 (18) Å) define a NS_4_ donor set. The coordination geometry is distorted towards square pyramidal (SP). This is quantified by the value of τ = 1/3, which compares with the ideal values of 0.0 and 1.0 for SP and TB, respectively (Addison *et al.*, 1984).

In the crystal structure, C—H···S contacts link molecules into a supramolecular chain, Table 1 and Fig. 2. Chains are linked into a 2-D array *via* C—H···π contacts where the π-system is defined by the ZnS_2_C chelate ring containing the S3 atom [C13—H13*a*···*Cg* = 2.86 Å, C13···*Cg* = 3.630 (3) Å with an angle of 136° at the H13*a* atom for symmetry operation 1 - *x*, 1 - *y*, -*z*].

### Experimental

Compound (I) was prepared by following a standard literature procedure (Lai & Tiekink, 2003) whereby two equivalents of Zn(S_2_CN(iPr)_2_)_2_ were added to *trans*-1,2-bis(4-pyridyl)ethylene.  Golden blocks of (I) were obtained from the slow evaporation of a chloroform/acetonitrile solution (3/1) of (I); m. pt. 513–515 K.

### Refinement

The H atoms were geometrically placed (C—H = 0.95–1.00 Å) and refined as riding with *U*_iso_(H) = 1.2–1.5*U*_eq_(C).

### Crystal data

**Table:**

[Zn_2_(C_7_H_14_NS_2_)_4_(C_12_H_10_N_2_)]	*Z* = 1
*M**_r_* = 1018.21	*F*(000) = 536
Triclinic, *P*1	*D*_x_ = 1.375 Mg m^−^^3^
Hall symbol: -P 1	Mo *K*α radiation, λ = 0.71069 Å
*a* = 8.2690 (14) Å	Cell parameters from 4206 reflections
*b* = 11.1640 (18) Å	θ = 2.6–40.2°
*c* = 14.156 (2) Å	µ = 1.35 mm^−^^1^
α = 80.806 (10)°	*T* = 98 K
β = 84.878 (9)°	Block, gold
γ = 72.566 (5)°	0.43 × 0.35 × 0.22 mm
*V* = 1229.6 (3) Å^3^	

### Data collection

**Table:**

Rigaku AFC12K/SATURN724 diffractometer	5613 independent reflections
Radiation source: fine-focus sealed tube	5323 reflections with *I* > 2σ(*I*)
graphite	*R*_int_ = 0.027
ω scans	θ_max_ = 27.5°, θ_min_ = 2.3°
Absorption correction: multi-scan (*ABSCOR*; Higashi, 1995)	*h* = −9→10
*T*_min_ = 0.810, *T*_max_ = 1	*k* = −13→14
9453 measured reflections	*l* = −18→18

### Refinement

**Table:**

Refinement on *F*^2^	Primary atom site location: structure-invariant direct methods
Least-squares matrix: full	Secondary atom site location: difference Fourier map
*R*[*F*^2^ > 2σ(*F*^2^)] = 0.038	Hydrogen site location: inferred from neighbouring sites
*wR*(*F*^2^) = 0.103	H-atom parameters constrained
*S* = 1.08	*w* = 1/[σ^2^(*F*_o_^2^) + (0.0536*P*)^2^ + 0.8556*P*] where *P* = (*F*_o_^2^ + 2*F*_c_^2^)/3
5613 reflections	(Δ/σ)_max_ = 0.001
261 parameters	Δρ_max_ = 0.73 e Å^−^^3^
0 restraints	Δρ_min_ = −0.93 e Å^−^^3^

### Special details

**Table:**

Geometry. All s.u.'s (except the s.u. in the dihedral angle between two l.s. planes) are estimated using the full covariance matrix. The cell s.u.'s are taken into account individually in the estimation of s.u.'s in distances, angles and torsion angles; correlations between s.u.'s in cell parameters are only used when they are defined by crystal symmetry. An approximate (isotropic) treatment of cell s.u.'s is used for estimating s.u.'s involving l.s. planes.
Refinement. Refinement of *F*^2^ against ALL reflections. The weighted *R*-factor *wR* and goodness of fit *S* are based on *F*^2^, conventional *R*-factors *R* are based on *F*, with *F* set to zero for negative *F*^2^. The threshold expression of *F*^2^ > 2σ(*F*^2^) is used only for calculating *R*-factors(gt) *etc*. and is not relevant to the choice of reflections for refinement. *R*-factors based on *F*^2^ are statistically about twice as large as those based on *F*, and *R*- factors based on ALL data will be even larger.

### Fractional atomic coordinates and isotropic or equivalent isotropic displacement parameters (Å^2^)

**Table:**

	*x*	*y*	*z*	*U*_iso_*/*U*_eq_	
Zn	0.26250 (3)	0.37511 (2)	0.262741 (17)	0.01954 (9)	
S1	0.50382 (7)	0.25806 (5)	0.35054 (4)	0.02363 (13)	
S2	0.31926 (7)	0.14535 (5)	0.24038 (4)	0.02353 (13)	
S3	0.18968 (7)	0.47710 (5)	0.10606 (4)	0.02045 (12)	
S4	0.29663 (7)	0.59904 (5)	0.24745 (4)	0.02168 (12)	
N1	0.6241 (2)	0.01811 (18)	0.31035 (14)	0.0237 (4)	
N2	0.2671 (2)	0.69764 (16)	0.06127 (12)	0.0191 (3)	
N3	0.0406 (2)	0.39861 (17)	0.34610 (13)	0.0203 (3)	
C1	0.4978 (3)	0.1269 (2)	0.30131 (15)	0.0193 (4)	
C2	0.7810 (3)	−0.0048 (2)	0.36315 (18)	0.0302 (5)	
H2	0.8458	−0.0952	0.3587	0.036*	
C3	0.7483 (4)	0.0018 (3)	0.46940 (19)	0.0377 (6)	
H3A	0.7019	0.0906	0.4798	0.057*	
H3B	0.8549	−0.0362	0.5023	0.057*	
H3C	0.6668	−0.0448	0.4949	0.057*	
C4	0.8972 (3)	0.0716 (3)	0.3145 (2)	0.0356 (6)	
H4A	0.9121	0.0632	0.2462	0.053*	
H4B	1.0078	0.0397	0.3438	0.053*	
H4C	0.8467	0.1610	0.3222	0.053*	
C5	0.6116 (3)	−0.0926 (2)	0.26693 (19)	0.0300 (5)	
H5	0.5131	−0.0596	0.2243	0.036*	
C6	0.7657 (4)	−0.1476 (3)	0.2036 (2)	0.0455 (7)	
H6A	0.7888	−0.0796	0.1564	0.068*	
H6B	0.7436	−0.2112	0.1703	0.068*	
H6C	0.8642	−0.1874	0.2432	0.068*	
C7	0.5682 (6)	−0.1896 (3)	0.3430 (2)	0.0585 (10)	
H7A	0.6630	−0.2270	0.3856	0.088*	
H7B	0.5470	−0.2563	0.3129	0.088*	
H7C	0.4664	−0.1490	0.3802	0.088*	
C8	0.2528 (2)	0.60365 (19)	0.12971 (14)	0.0173 (4)	
C9	0.3395 (3)	0.7973 (2)	0.08196 (16)	0.0230 (4)	
H9	0.3809	0.7714	0.1485	0.028*	
C10	0.4919 (3)	0.8049 (3)	0.0156 (2)	0.0356 (6)	
H10A	0.5762	0.7213	0.0199	0.053*	
H10B	0.5422	0.8662	0.0346	0.053*	
H10C	0.4558	0.8323	−0.0504	0.053*	
C11	0.2042 (3)	0.9242 (2)	0.0808 (2)	0.0342 (6)	
H11A	0.1617	0.9535	0.0162	0.051*	
H11B	0.2532	0.9863	0.0996	0.051*	
H11C	0.1105	0.9147	0.1260	0.051*	
C12	0.2101 (3)	0.7146 (2)	−0.03856 (15)	0.0230 (4)	
H12	0.2300	0.7958	−0.0707	0.028*	
C13	0.3156 (3)	0.6133 (2)	−0.09784 (17)	0.0302 (5)	
H13A	0.4362	0.5970	−0.0873	0.045*	
H13B	0.2948	0.6428	−0.1659	0.045*	
H13C	0.2835	0.5349	−0.0785	0.045*	
C14	0.0198 (3)	0.7356 (2)	−0.04095 (18)	0.0271 (5)	
H14A	−0.0069	0.6565	−0.0153	0.041*	
H14B	−0.0155	0.7621	−0.1072	0.041*	
H14C	−0.0405	0.8017	−0.0019	0.041*	
C15	0.0365 (3)	0.3549 (2)	0.44008 (17)	0.0294 (5)	
H15	0.1404	0.3103	0.4696	0.035*	
C16	−0.1129 (3)	0.3724 (2)	0.49564 (16)	0.0297 (5)	
H16	−0.1101	0.3407	0.5621	0.036*	
C17	−0.2677 (3)	0.4365 (2)	0.45415 (15)	0.0200 (4)	
C18	−0.2633 (3)	0.4766 (2)	0.35583 (15)	0.0224 (4)	
H18	−0.3658	0.5165	0.3236	0.027*	
C19	−0.1086 (3)	0.4576 (2)	0.30552 (16)	0.0228 (4)	
H19	−0.1078	0.4879	0.2389	0.027*	
C20	−0.4262 (3)	0.4592 (2)	0.51333 (15)	0.0207 (4)	
H20	−0.4230	0.4119	0.5755	0.025*	

### Atomic displacement parameters (Å^2^)

**Table:**

	*U*^11^	*U*^22^	*U*^33^	*U*^12^	*U*^13^	*U*^23^
Zn	0.01645 (14)	0.02258 (14)	0.01769 (14)	−0.00523 (10)	0.00223 (9)	0.00022 (9)
S1	0.0225 (3)	0.0227 (3)	0.0275 (3)	−0.0087 (2)	−0.0054 (2)	−0.0022 (2)
S2	0.0220 (3)	0.0234 (3)	0.0256 (3)	−0.0055 (2)	−0.0057 (2)	−0.0041 (2)
S3	0.0237 (3)	0.0223 (2)	0.0171 (2)	−0.0098 (2)	0.00010 (19)	−0.00222 (18)
S4	0.0266 (3)	0.0246 (3)	0.0151 (2)	−0.0099 (2)	−0.0018 (2)	−0.00120 (18)
N1	0.0215 (9)	0.0222 (9)	0.0260 (10)	−0.0062 (7)	−0.0042 (7)	0.0016 (7)
N2	0.0201 (8)	0.0203 (8)	0.0158 (8)	−0.0052 (7)	−0.0011 (7)	−0.0008 (6)
N3	0.0180 (8)	0.0230 (8)	0.0184 (9)	−0.0055 (7)	0.0028 (7)	−0.0010 (7)
C1	0.0183 (9)	0.0232 (10)	0.0165 (9)	−0.0084 (8)	0.0013 (7)	0.0006 (7)
C2	0.0224 (11)	0.0340 (12)	0.0316 (13)	−0.0067 (9)	−0.0065 (9)	0.0032 (10)
C3	0.0390 (14)	0.0450 (15)	0.0295 (13)	−0.0128 (12)	−0.0069 (11)	−0.0022 (11)
C4	0.0227 (11)	0.0472 (15)	0.0392 (14)	−0.0146 (11)	−0.0009 (10)	−0.0041 (11)
C5	0.0306 (12)	0.0234 (11)	0.0343 (13)	−0.0040 (9)	−0.0037 (10)	−0.0054 (9)
C6	0.0463 (17)	0.0485 (16)	0.0402 (16)	−0.0061 (13)	0.0026 (13)	−0.0187 (13)
C7	0.099 (3)	0.0461 (18)	0.0459 (18)	−0.0458 (19)	0.0196 (18)	−0.0164 (14)
C8	0.0153 (9)	0.0202 (9)	0.0151 (9)	−0.0037 (7)	0.0008 (7)	−0.0028 (7)
C9	0.0254 (11)	0.0218 (10)	0.0234 (11)	−0.0098 (8)	−0.0024 (8)	−0.0015 (8)
C10	0.0341 (13)	0.0345 (13)	0.0452 (15)	−0.0209 (11)	0.0083 (11)	−0.0099 (11)
C11	0.0288 (12)	0.0276 (12)	0.0483 (16)	−0.0057 (10)	−0.0019 (11)	−0.0162 (11)
C12	0.0271 (11)	0.0239 (10)	0.0169 (10)	−0.0066 (8)	−0.0036 (8)	−0.0001 (8)
C13	0.0356 (13)	0.0348 (12)	0.0190 (11)	−0.0075 (10)	0.0027 (9)	−0.0072 (9)
C14	0.0249 (11)	0.0269 (11)	0.0293 (12)	−0.0056 (9)	−0.0076 (9)	−0.0038 (9)
C15	0.0194 (10)	0.0407 (13)	0.0209 (11)	−0.0023 (9)	−0.0004 (9)	0.0043 (9)
C16	0.0209 (11)	0.0438 (14)	0.0174 (11)	−0.0035 (10)	0.0020 (9)	0.0036 (9)
C17	0.0182 (10)	0.0221 (10)	0.0209 (10)	−0.0081 (8)	0.0017 (8)	−0.0031 (8)
C18	0.0175 (10)	0.0281 (11)	0.0187 (10)	−0.0038 (8)	0.0008 (8)	−0.0014 (8)
C19	0.0192 (10)	0.0289 (11)	0.0183 (10)	−0.0062 (8)	0.0013 (8)	−0.0002 (8)
C20	0.0197 (10)	0.0262 (10)	0.0162 (9)	−0.0075 (8)	0.0031 (8)	−0.0034 (8)

### Geometric parameters (Å, °)

**Table:**

Zn—N3	2.0621 (18)	C7—H7A	0.9800
Zn—S1	2.3655 (7)	C7—H7B	0.9800
Zn—S3	2.3662 (7)	C7—H7C	0.9800
Zn—S2	2.5320 (7)	C9—C10	1.518 (3)
Zn—S4	2.5720 (7)	C9—C11	1.519 (3)
S1—C1	1.734 (2)	C9—H9	1.0000
S2—C1	1.719 (2)	C10—H10A	0.9800
S3—C8	1.733 (2)	C10—H10B	0.9800
S4—C8	1.727 (2)	C10—H10C	0.9800
N1—C1	1.340 (3)	C11—H11A	0.9800
N1—C2	1.490 (3)	C11—H11B	0.9800
N1—C5	1.499 (3)	C11—H11C	0.9800
N2—C8	1.335 (3)	C12—C14	1.522 (3)
N2—C9	1.489 (3)	C12—C13	1.525 (3)
N2—C12	1.495 (3)	C12—H12	1.0000
N3—C15	1.343 (3)	C13—H13A	0.9800
N3—C19	1.344 (3)	C13—H13B	0.9800
C2—C3	1.514 (4)	C13—H13C	0.9800
C2—C4	1.520 (4)	C14—H14A	0.9800
C2—H2	1.0000	C14—H14B	0.9800
C3—H3A	0.9800	C14—H14C	0.9800
C3—H3B	0.9800	C15—C16	1.384 (3)
C3—H3C	0.9800	C15—H15	0.9500
C4—H4A	0.9800	C16—C17	1.395 (3)
C4—H4B	0.9800	C16—H16	0.9500
C4—H4C	0.9800	C17—C18	1.393 (3)
C5—C7	1.501 (4)	C17—C20	1.469 (3)
C5—C6	1.519 (4)	C18—C19	1.383 (3)
C5—H5	1.0000	C18—H18	0.9500
C6—H6A	0.9800	C19—H19	0.9500
C6—H6B	0.9800	C20—C20^i^	1.330 (4)
C6—H6C	0.9800	C20—H20	0.9500
			
N3—Zn—S1	112.29 (5)	H7A—C7—H7C	109.5
N3—Zn—S3	107.48 (5)	H7B—C7—H7C	109.5
S1—Zn—S3	140.23 (2)	N2—C8—S4	122.56 (15)
N3—Zn—S2	99.44 (5)	N2—C8—S3	122.09 (15)
S1—Zn—S2	73.21 (2)	S4—C8—S3	115.36 (12)
S3—Zn—S2	100.61 (2)	N2—C9—C10	111.56 (18)
N3—Zn—S4	100.23 (5)	N2—C9—C11	111.17 (18)
S1—Zn—S4	99.98 (2)	C10—C9—C11	112.7 (2)
S3—Zn—S4	72.47 (2)	N2—C9—H9	107.0
S2—Zn—S4	160.31 (2)	C10—C9—H9	107.0
C1—S1—Zn	87.45 (7)	C11—C9—H9	107.0
C1—S2—Zn	82.55 (7)	C9—C10—H10A	109.5
C8—S3—Zn	88.80 (7)	C9—C10—H10B	109.5
C8—S4—Zn	82.47 (7)	H10A—C10—H10B	109.5
C1—N1—C2	124.9 (2)	C9—C10—H10C	109.5
C1—N1—C5	119.88 (19)	H10A—C10—H10C	109.5
C2—N1—C5	115.18 (19)	H10B—C10—H10C	109.5
C8—N2—C9	120.53 (17)	C9—C11—H11A	109.5
C8—N2—C12	124.50 (18)	C9—C11—H11B	109.5
C9—N2—C12	114.95 (16)	H11A—C11—H11B	109.5
C15—N3—C19	117.40 (19)	C9—C11—H11C	109.5
C15—N3—Zn	123.08 (15)	H11A—C11—H11C	109.5
C19—N3—Zn	119.50 (14)	H11B—C11—H11C	109.5
N1—C1—S2	122.16 (16)	N2—C12—C14	112.12 (18)
N1—C1—S1	122.17 (16)	N2—C12—C13	113.96 (18)
S2—C1—S1	115.67 (12)	C14—C12—C13	113.47 (19)
N1—C2—C3	113.7 (2)	N2—C12—H12	105.4
N1—C2—C4	113.0 (2)	C14—C12—H12	105.4
C3—C2—C4	114.1 (2)	C13—C12—H12	105.4
N1—C2—H2	104.9	C12—C13—H13A	109.5
C3—C2—H2	104.9	C12—C13—H13B	109.5
C4—C2—H2	104.9	H13A—C13—H13B	109.5
C2—C3—H3A	109.5	C12—C13—H13C	109.5
C2—C3—H3B	109.5	H13A—C13—H13C	109.5
H3A—C3—H3B	109.5	H13B—C13—H13C	109.5
C2—C3—H3C	109.5	C12—C14—H14A	109.5
H3A—C3—H3C	109.5	C12—C14—H14B	109.5
H3B—C3—H3C	109.5	H14A—C14—H14B	109.5
C2—C4—H4A	109.5	C12—C14—H14C	109.5
C2—C4—H4B	109.5	H14A—C14—H14C	109.5
H4A—C4—H4B	109.5	H14B—C14—H14C	109.5
C2—C4—H4C	109.5	N3—C15—C16	122.7 (2)
H4A—C4—H4C	109.5	N3—C15—H15	118.7
H4B—C4—H4C	109.5	C16—C15—H15	118.7
N1—C5—C7	110.4 (2)	C15—C16—C17	120.0 (2)
N1—C5—C6	113.3 (2)	C15—C16—H16	120.0
C7—C5—C6	113.4 (3)	C17—C16—H16	120.0
N1—C5—H5	106.4	C18—C17—C16	117.1 (2)
C7—C5—H5	106.4	C18—C17—C20	122.64 (19)
C6—C5—H5	106.4	C16—C17—C20	120.3 (2)
C5—C6—H6A	109.5	C19—C18—C17	119.4 (2)
C5—C6—H6B	109.5	C19—C18—H18	120.3
H6A—C6—H6B	109.5	C17—C18—H18	120.3
C5—C6—H6C	109.5	N3—C19—C18	123.3 (2)
H6A—C6—H6C	109.5	N3—C19—H19	118.3
H6B—C6—H6C	109.5	C18—C19—H19	118.3
C5—C7—H7A	109.5	C20^i^—C20—C17	125.1 (3)
C5—C7—H7B	109.5	C20^i^—C20—H20	117.4
H7A—C7—H7B	109.5	C17—C20—H20	117.4
C5—C7—H7C	109.5		
			
N3—Zn—S1—C1	99.95 (9)	C1—N1—C2—C4	−68.5 (3)
S3—Zn—S1—C1	−80.02 (8)	C5—N1—C2—C4	112.6 (2)
S2—Zn—S1—C1	6.43 (7)	C1—N1—C5—C7	−103.7 (3)
S4—Zn—S1—C1	−154.56 (7)	C2—N1—C5—C7	75.3 (3)
N3—Zn—S2—C1	−117.11 (9)	C1—N1—C5—C6	128.0 (2)
S1—Zn—S2—C1	−6.53 (7)	C2—N1—C5—C6	−53.1 (3)
S3—Zn—S2—C1	132.96 (7)	C9—N2—C8—S4	6.4 (3)
S4—Zn—S2—C1	65.65 (9)	C12—N2—C8—S4	−171.92 (16)
N3—Zn—S3—C8	101.33 (9)	C9—N2—C8—S3	−173.29 (15)
S1—Zn—S3—C8	−78.70 (7)	C12—N2—C8—S3	8.4 (3)
S2—Zn—S3—C8	−155.14 (7)	Zn—S4—C8—N2	−171.20 (18)
S4—Zn—S3—C8	5.83 (7)	Zn—S4—C8—S3	8.52 (10)
N3—Zn—S4—C8	−111.15 (9)	Zn—S3—C8—N2	170.53 (17)
S1—Zn—S4—C8	133.82 (7)	Zn—S3—C8—S4	−9.19 (11)
S3—Zn—S4—C8	−5.90 (7)	C8—N2—C9—C10	122.8 (2)
S2—Zn—S4—C8	66.08 (9)	C12—N2—C9—C10	−58.7 (2)
S1—Zn—N3—C15	1.8 (2)	C8—N2—C9—C11	−110.5 (2)
S3—Zn—N3—C15	−178.20 (18)	C12—N2—C9—C11	67.9 (2)
S2—Zn—N3—C15	77.44 (19)	C8—N2—C12—C14	61.8 (3)
S4—Zn—N3—C15	−103.51 (19)	C9—N2—C12—C14	−116.6 (2)
S1—Zn—N3—C19	−176.66 (15)	C8—N2—C12—C13	−68.8 (3)
S3—Zn—N3—C19	3.32 (17)	C9—N2—C12—C13	112.8 (2)
S2—Zn—N3—C19	−101.04 (16)	C19—N3—C15—C16	−2.2 (4)
S4—Zn—N3—C19	78.01 (16)	Zn—N3—C15—C16	179.3 (2)
C2—N1—C1—S2	−179.16 (17)	N3—C15—C16—C17	0.6 (4)
C5—N1—C1—S2	−0.3 (3)	C15—C16—C17—C18	2.3 (4)
C2—N1—C1—S1	0.7 (3)	C15—C16—C17—C20	−177.9 (2)
C5—N1—C1—S1	179.53 (17)	C16—C17—C18—C19	−3.5 (3)
Zn—S2—C1—N1	−170.64 (18)	C20—C17—C18—C19	176.7 (2)
Zn—S2—C1—S1	9.49 (10)	C15—N3—C19—C18	0.9 (3)
Zn—S1—C1—N1	170.04 (18)	Zn—N3—C19—C18	179.48 (17)
Zn—S1—C1—S2	−10.09 (11)	C17—C18—C19—N3	2.0 (4)
C1—N1—C2—C3	63.5 (3)	C18—C17—C20—C20^i^	−15.2 (4)
C5—N1—C2—C3	−115.3 (2)	C16—C17—C20—C20^i^	164.9 (3)

Symmetry codes: (i) −*x*−1, −*y*+1, −*z*+1.

### Hydrogen-bond geometry (Å, °)

**Table:**

*D*—H···*A*	*D*—H	H···*A*	*D*···*A*	*D*—H···*A*
C20—H20···S4^ii^	0.95	2.77	3.545 (2)	139

Symmetry codes: (ii) −*x*, −*y*+1, −*z*+1.
